# Vaginoplasty tips and tricks

**DOI:** 10.1590/S1677-5538.IBJU.2020.0338

**Published:** 2021-02-03

**Authors:** Joy S. Li, Curtis N. Crane, Richard A. Santucci

**Affiliations:** 1 University of Texas Austin US University of Texas at Austin, 110 Inner Campus Drive, Austin, US.; 2 Crane Center for Transgender Surgery Austin US Crane Center for Transgender Surgery, Austin, US.

**Keywords:** Sex Reassignment Surgery, Gender Dysphoria, Surgical Procedures, Operative

## Abstract

Vaginoplasty is a commonly performed surgery for the transfeminine patient. In this review, we discuss how to achieve satisfactory surgical outcomes, and highlight solutions to common complications involved with the surgery, including: wound separation, vaginal stenosis, hematoma, and rectovaginal fistula. Pre-operative evaluation and standard technique are outlined. Goal outcomes regarding aesthetics, creation of a neocavity, urethral management, labial appearance, vaginal packing and clitoral sizing are all described. Peritoneal vaginoplasty technique and visceral interposition technique are detailed as alternatives to the penile inversion technique in case they are needed to be used. Post-operative patient satisfaction, patient care plans, and solutions to common complications are reviewed.

## OVERVIEW

Vaginoplasty is the most commonly performed surgery for gender affirmation, with more than 3.000 performed annually worldwide. Overall, it is a safe and reliable surgery for genital reconstruction in a male-to-female patient (1). Techniques include the most commonly used procedures: penile inversion, as well as visceral interposition and pelvic peritoneal vaginoplasty. Uncommon, but important complications include rectovaginal fistula and vaginal stenosis.

With greater social and medical acknowledgement of gender dysphoria, a wider range of patients are seeking care, including elderly, medically comorbid, and younger patients (who may require different vaginoplasty techniques and considerations because of the use of puberty blockers). Surgical care for transgender patients has steadily improved, and increased global experience has steadily enhanced outcomes. However, it is crucial to continue to improve these techniques for still better outcomes including improving the aesthetics and function of the neovagina, increasing patient satisfaction, and decreasing complication rates. At our high-volume center, we perform more than 140 vaginoplasty surgeries per year, and have an ongoing program of surgical improvement aimed at optimizing this surgery.

## INTRODUCTION

Approximately 44 million individuals worldwide are diagnosed with gender dysphoria, and with increased advocacy, acceptance, and access to care, individuals seeking care for gender dysphoria are likely to rise in the future (2). Gender affirmation surgery (GAS) has been demonstrated as the most effective treatment for gender dysphoria in those who desire it (3). Successful GAS relies on elements of psychotherapy, hormonal therapy, genital and non-genital surgical procedures (3). More specifically, transfeminine genital GAS (gGAS) refers to feminizing genital procedures including vaginoplasty. A detailed understanding of pelvic anatomy and specific understanding of patient's surgical goals are necessary for successful vaginoplasty.

Prior to surgery, the World Professional Association for Transgender Health (WPATH) guidelines should be considered. These guidelines are from the Standards of Care for the Health of Trans-sexual, Transgender, and Gender Non-conforming People, and serve to help determine appropriate candidates for surgery (4). Even though there are few absolute medical contraindications to surgery, the preoperative consultation should be used to identify relative risk factors associated with higher rates of complications, and to optimize care for such factors pre-operatively. It is crucial that the irreversibility of the procedure is emphasized, and mandatory to determine if the patient can safely undergo surgery and cope with the psychiatric and self-dilation demands that their new anatomy will produce.

The type of vaginoplasty performed is dependent on every individual's specific needs and surgical goals. Some patients may choose a “zero––depth” vaginoplasty (aka “vulvoplasty”) if they do not anticipate to ever want vaginal penetration, or they may have comorbidities that make this a safer option (5). Younger patients in whom puberty has been arrested, may have insufficient penile skin needed for neovaginal cavity augmentation in excess of what is available through standard penile inversion techniques (2). To accommodate diverse patient needs, high volume centers offering vaginoplasty should be well-versed in more than one technique and offer alternatives when appropriate.

In this review, we discuss how to achieve satisfactory surgical outcomes and highlight solutions to common complications associated with vaginoplasty. The anatomy, as well as aesthetic and functional parameters, including the different techniques involved in the creation of a canal, are discussed. In addition, the penile inversion technique is detailed because it is the usual technique of choice for vaginoplasty. The visceral interposition and peritoneal vaginoplasty techniques are also briefly outlined as secondary methods to create the neovaginal cavity. Finally, methods to maximize patient satisfaction and solutions for common complications, including rectovaginal fistula and vaginal stenosis, are summarized.

### Pre-operative medical care

After receiving WPATH support letters and completing depilation (if desired), surgery is scheduled. Notably, preoperative hair removal is only required at the proximal shaft of the phallus, as this tissue will be transferred to the vaginal cavity and will continue to grow hair after vaginoplasty. Patients with chronic medical conditions such as hypertension, diabetes and/or hypercoagulable state are cleared and optimized by the patient's own physicians. The day before the surgery, the patient completes a mechanical colon prep.

### BMI and vaginoplasty

A thorough evaluation of the thousands of manuscripts that discuss the role of body weight and surgical complications is beyond the scope of this paper. Many surgery centers have a BMI cutoff over which gender confirmation surgery is not performed, which varies from center to center. At our center there is no cutoff for feminizing mastectomy for example, but we do have a strong preference for patients to have a BMI of 39 or lower for vaginoplasty. Many other centers have a more restrictive cut-off.

The literature seems clear that as BMI increases by decile (i.e.: from 20's to the 30's), overall complication rate rises predictably (6, 7). Some of these complications are self-limited wound infections, but other notable complications, such increased, possibly deadly venous thromboembolism (VTE), have also been reported (6). Our practice is to assess the patient's general health and cardiac risk first and foremost. While rising BMI has an association with diabetes, cardiovascular disease, hypertension, obstructive sleep apnea and surgical infection, each of these risks must be independently assessed as being present/not present in each patient regardless of BMI. Patients with higher risk require thorough optimization and surgical clearance from their primary care physicians and medical specialists. Patients with diabetes should have excellent control of blood sugar, and we prefer pre-operative HgA1c values below 7.0.

### Standard Inversion Technique

Penile inversion vaginoplasty is the most common vaginoplasty technique utilized today (2).

**Figure 1:**
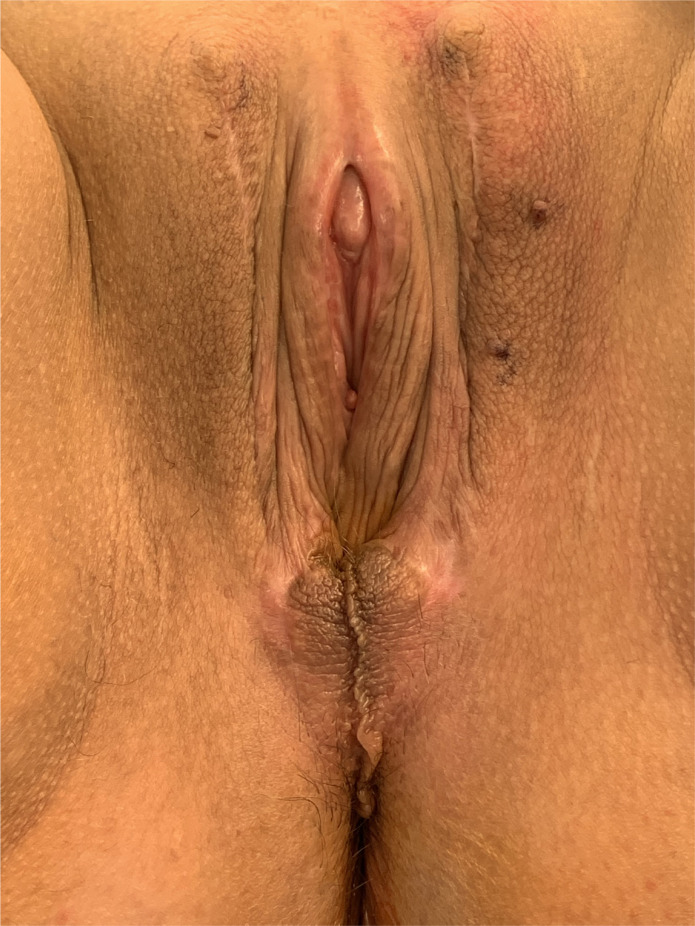
Well-healed postoperative appearance after penile inversion vaginoplasty showing excellent appearance of labia majora, labia minora and clitoris. Note the appearance of the clitoral hood, and the well-sized clitoris, which closely mirrors that found in the natal female.

From an embryologic perspective, understanding homologous structures between male and female genitalia creates a framework for both the deconstructive and reconstructive approach to the surgery. The labia majora are formed from the lateral periscrotal skin, the labia minora are created from lateral neovaginal tissue (former penile base skin), and the clitoris is formed from the reduced dorsal glans. The neoclitoris is perfused by the dorsal penile artery and is innervated by its dorsal penile nerve and so requires that the neurovascular bundle be meticulously preserved. To allow for the creation of the inner labia minora, urethral meatus, and vestibular lining, the penile urethra is shortened, spatulated and everted. To augment the vaginal depth, full-thickness skin grafts can be harvested from scrotum and sewn at the end of the inverted penile skin sleeve (8). This technique creates an aesthetic and functional vagina and external genitalia (Figure-1).

### Brief step-by-step of the standard inversion technique

**Figure 2:**
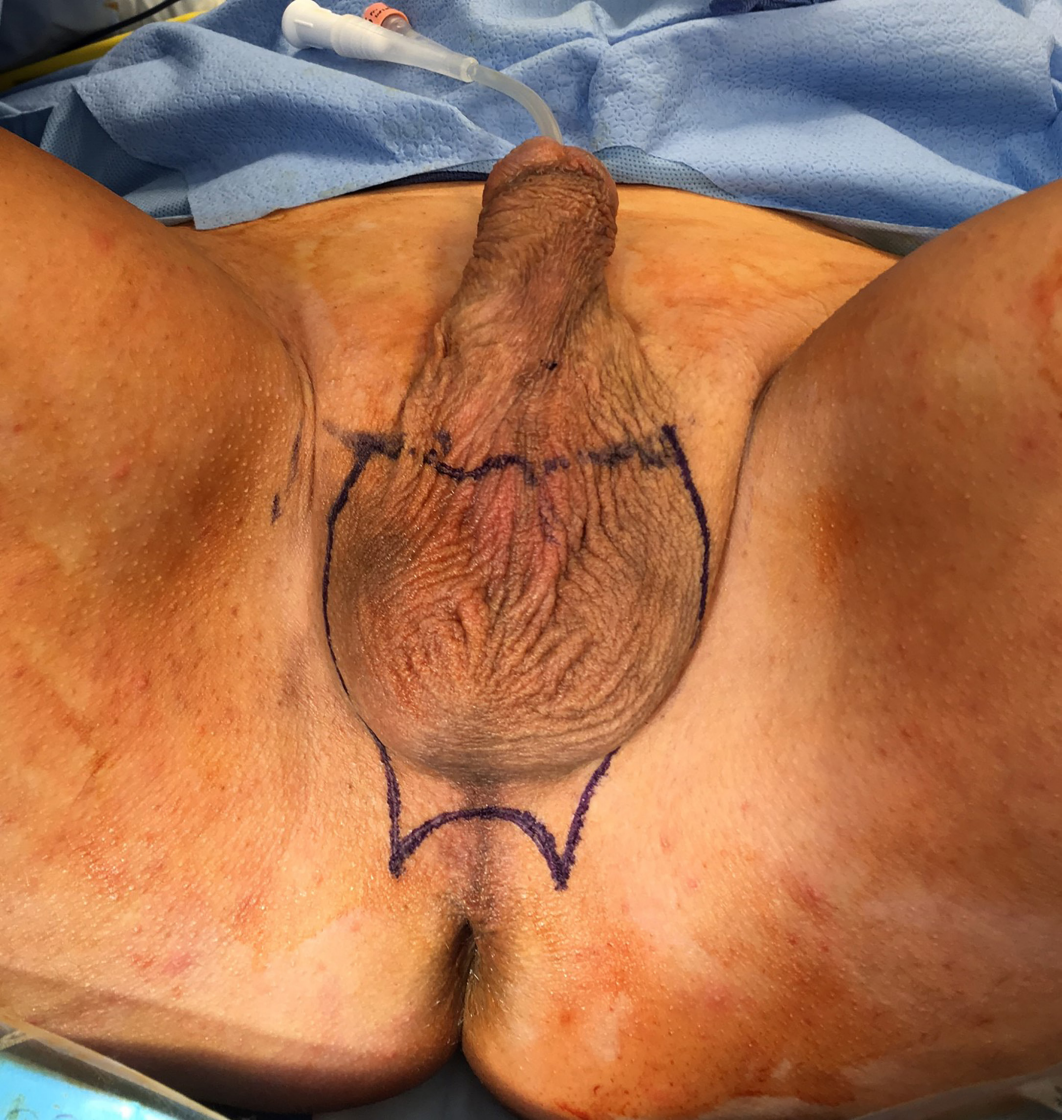
Surgical markings indicating the borders of the scrotal skin removal, sparing the penile base tissue, and creating a wide-open perineal opening for the future vagina.

**Figure 3:**
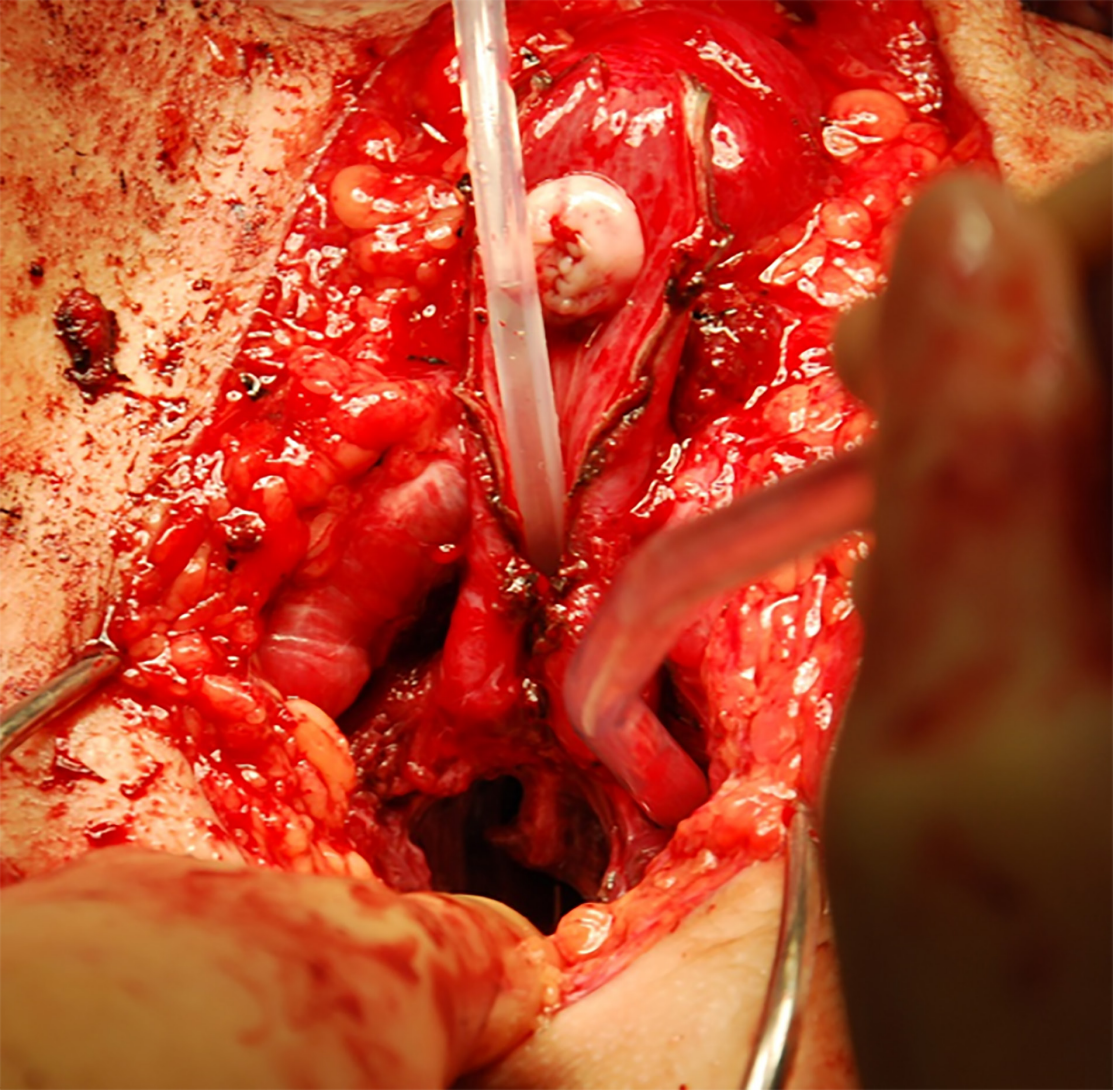
A neovaginal space at least 14cm deep and 6cm wide is created. In this photo, a second team has made the clitoris already.

**Figure 4:**
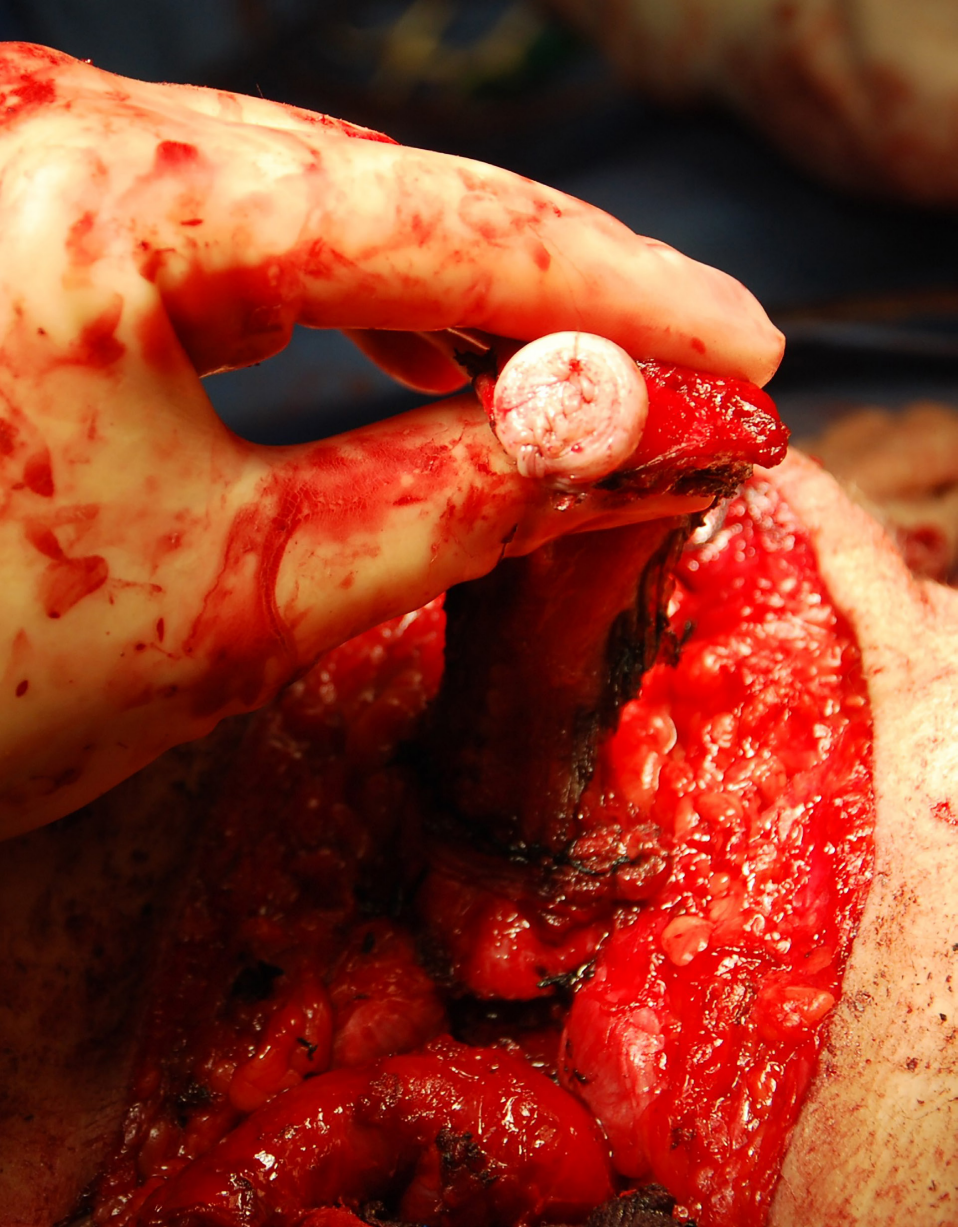
An aesthetic, properly sized clitoris is made from the dorsal glans. Note preservation of the nerve, artery, and vein of the phallus including the tunica albuginea over which it courses.

**Figure 5:**
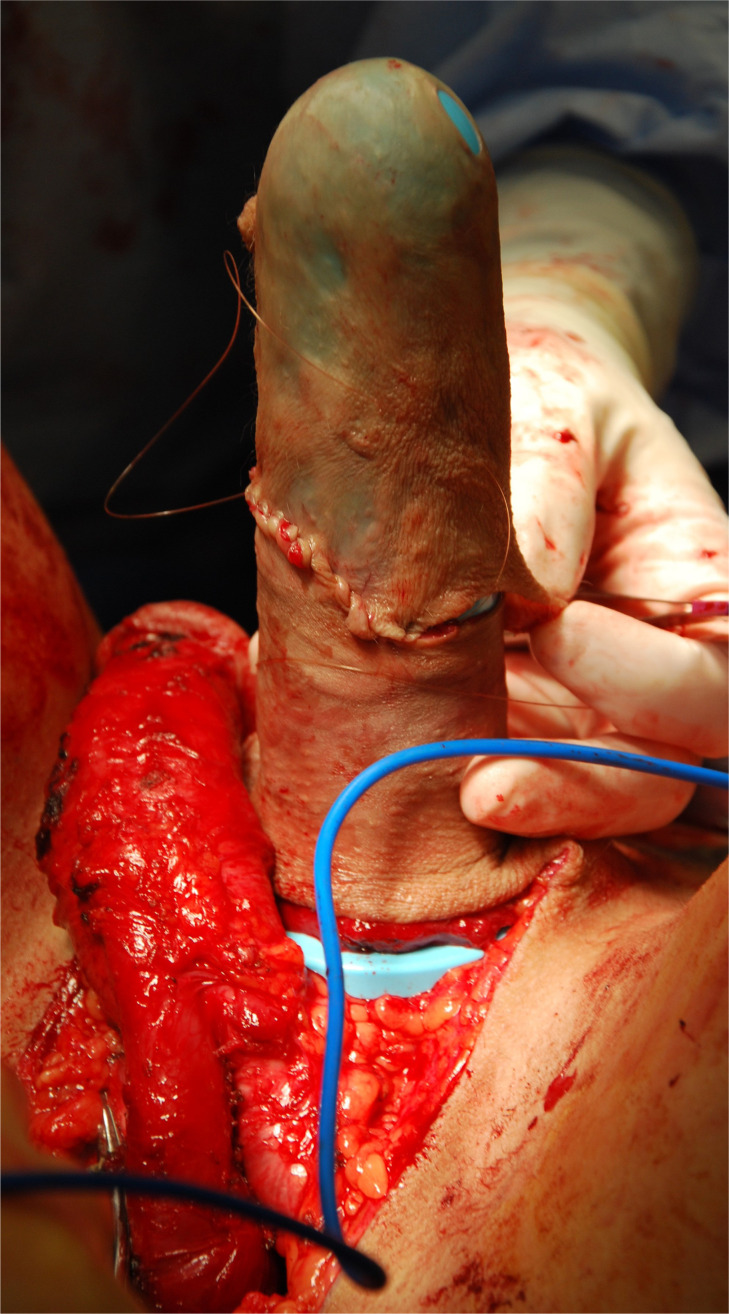
The penile skin flap is sewn to the thinned scrotal tissue, over a vaginal form, to create a large-size vaginal lining.

**Figure 6:**
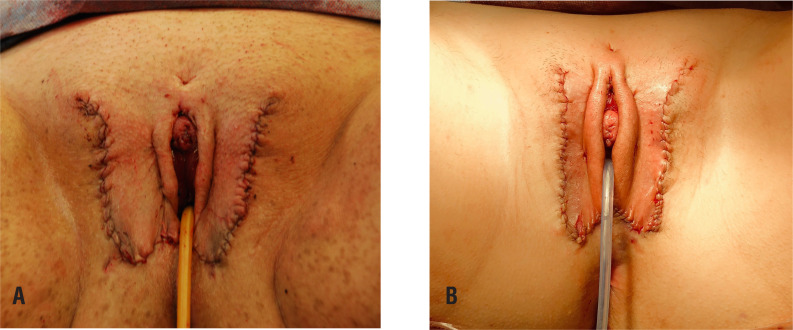
Two examples of immediate post op result. Note minor differences in the size and configuration of the external genitalia, just as in natal females.

**Figure 7:**
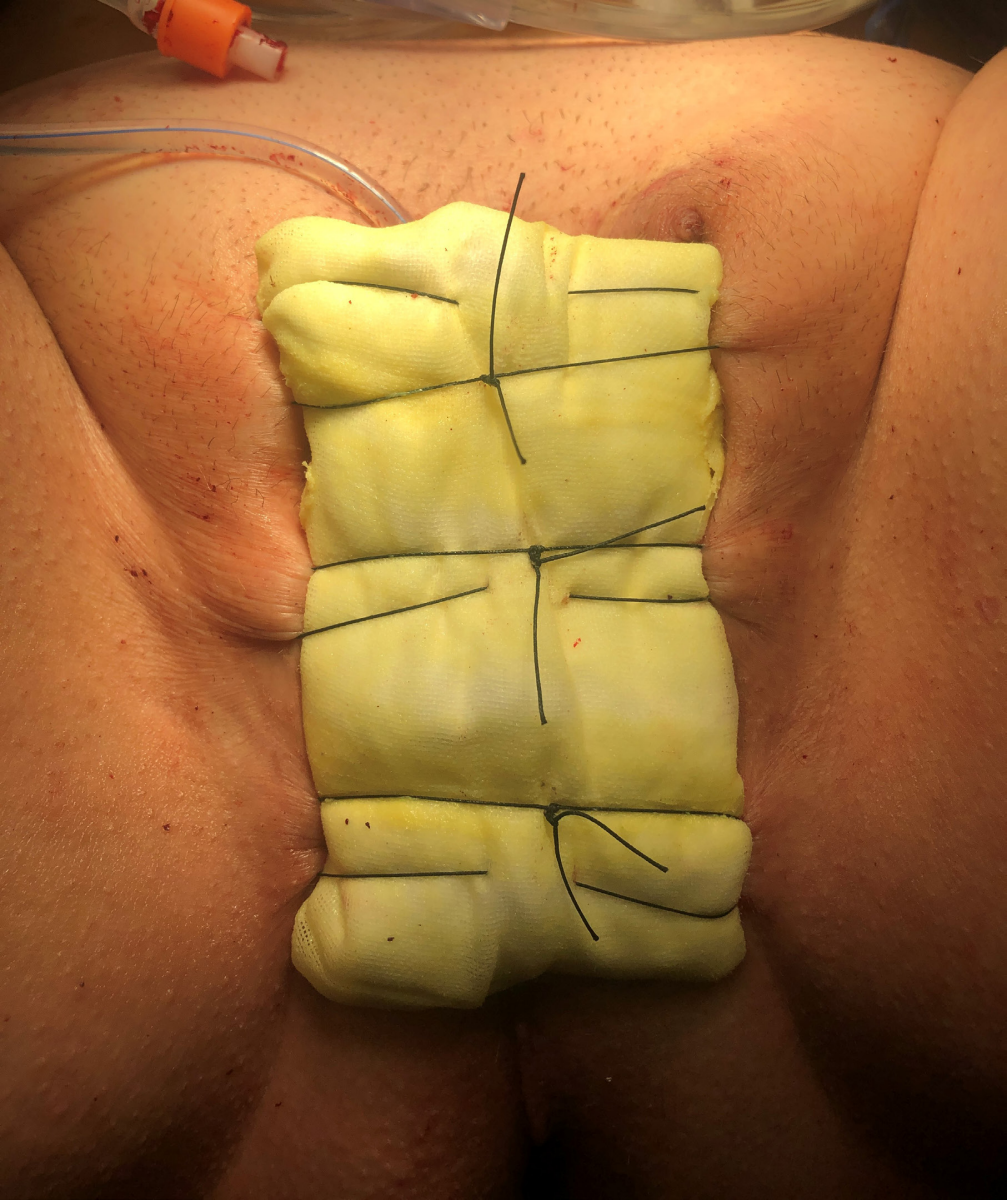
After vaginal packing is placed, a bolster dressing is placed over the packing to eliminate the possibility of packing extrusion. The bolster dressing can be extended over the entire genitals to provide light pressure to combat swelling and hematoma.

1. Identify and mark the perineal flap to indicate the location of the vaginal introitus. (Figure-2).
  - Excellent vaginal depth can be achieved without using any perineal skin flaps, which is desirable as this tissue can introduce hair bearing skin into the introitus. Also, sparing the skin of the perineum provides a useful “tissue bank” of healthy skin flaps that can be used later to repair those patients who might develop narrowing of the introitus.
2. To create the future limits of the vaginal introitus, mark the anterior limit of the incision by measuring from the perineal body to the penoscrotal junction along the perineal midline raphe so that the future labia majora will be smooth, graded and continuous appearing. This v-shaped design also reduces the risk of introital contracture.
3. After orchiectomy, create the vaginal canal within the space between the rectum and prostate (Figure-3). Use cautery to make a midline incision through the perineal fascia into the deep perineal pouch. Dissection continues to the peritoneal reflection until the cavity is at least 14cm deep. This is done by following Denonvillier's fascia to its terminus. While dissecting, avoid iatrogenic injury to the rectum, prostate, and bladder. Incise the ischiocavernosus muscles after the vaginal cavity is created to enlarge its width.
4. Resect the bulbospongiosus muscles (because of anecdotal reports of difficulty urinating with sexual arousal when the muscle is not removed).
5. Separate the urethra from the corpora and discard distal unneeded urethra. Partially remove the ventral corporal bodies, meticulously sparing the neurovascular bundle, its underlying corpora and the portion of the glans designated to make the neoclitoris.
6. Create a neoclitoris (Figure-4) and loosely affix the neurovascular pedicle to the fascia of the anterior abdominal wall to prevent migration.
7. Position the penile flap/scrotal skin complex into the vaginal cavity after suturing the penile and scrotal-perineal grafts together (Figure-5).
8. Invert and advance the flap into the neo-vagina.
  - The labia majora are created naturally when the penile skin is involuted, which only requires suturing of the skin edges to complete.
9. Construct the labia minora and clitoral hood (Figure-6).
10. Place vaginal packing into the vaginal cavity and place a bolster dressing (Figure-7). Remove the vaginal packing after 5-7 days.

## GOAL OUTCOMES

### Anatomy and aesthetics

The ultimate goal for gender reassignment surgery in transfeminine patients is to create external genitalia congruent to the natal female, a functional urethra, and vaginal depth sufficient for intercourse if desired. Overall, the ideal neova-gina should have an aesthetic female appearance, a sensate neoclitoris, and a vaginal cavity that is functionally specific for a patient's needs.

The vestibule must appear moist, and the clitoris and its hood should be in the superior aspect and midline of the neolabia minora construct, with the neourethra approximately two thirds the distance from the clitoris to the introitus. To prevent the clitoris from being placed too high, the neoclitoris must be positioned at the line joining the adductor longus tendon. To ensure a moist appearance of the vestibule and allow for some lubrication with arousal, the medial labia minora are created from lateral urethral tissue, and the neoclitoris is brought through the center of the urethral flap.

To ensure that the introitus, neovagina, labia minora, clitoris and its hood are hairless, complete hair removal of the penile shaft should be accomplished preoperatively either by electrolysis or laser hair removal pre-operatively. Only the pe-nile shaft needs to be treated. Depilation may take more than one year to complete, so this should be factored in when planning surgery. Effective hair removal will not permanently remove all potential for future hair growth and may only decrease the density of hair on treated skin. Our experience is that no hair removal is truly permanent, and hair regrowth may be a feature of any vaginoplasty.

To avoid increased risk of infection and damage to the subdermal plexus, which is required to keep the penile flaps alive, hair removal should not be performed within two weeks of surgery.

### Cavity

A neovaginal cavity to allow for penetrative sexual intercourse is important for many patients. The potential space to create this cavity is determined by the anatomy of the male perineum/pelvis. The maximum size of the cavity depends on the body habitus. Although there is no standard size for the creation of a neovaginal cavity, a depth less than 6cm is associated with an increase in the need for secondary revision surgery (9). In most cases, we create a cavity measuring at least 14cm by 6cm to reduce the need for revision surgery (9). Larger cavities, especially in tall or large patients, are routinely achieved. However, patients must follow a strict dilating regimen or the cavity will shrink post-operatively. To prevent loss of depth and width, post-operative management by dilations may need to be maintained for the patient's lifetime.

In general, other than meticulous surgical technique, there is no widely accepted or scientifically-proven method for improving the outcomes pertaining to the vaginal cavity. We and others have endeavored to place growth-factor-rich material in the cavity to improve outcomes. We currently use 100mg of Amniofill™ (MiMedx; Marrietta, Georgia, USA) sprinkled into the cavity before placing the inverted penile skin flap in place. AmnioFill™ is a sterile tissue matrix allograft derived from human amniotic membrane (harvested during scheduled Caesarian section surgery) that preserves active extracellular matrix proteins, growth factors, cytokines, and other specialty growth factors to help enhance healing (10, 11). More than 100 scientific reports support the use of dehydrated human amnion to improve healing and decrease scarring. We have anecdotally seen fewer vaginal wound disruptions with its use, yet further research is required to determine its effectiveness in vaginoplasty surgery.

### Urethral management

We use the PKS SEAL™ Open Forceps, part of the Advanced Bipolar PK System (Olympus; Center Valley, PA), to simultaneously incise and seal the ventral urethra. This is an impedance-controlled, bipolar vessel sealing system that seems to greatly decrease bleeding and oozing from the cut urethral edge (12). The distal urethra is discarded, the distal bulbar urethra is incised to create the paraclitoral moist/pink tissue, and the proximal bulbar urethra is left in place as the shortened, female-type urethra. It is important that the bulbar urethra is incised to a point where it is absolutely straight from the bladder, as otherwise the urine may shoot up and over the toilet brim with seated voiding. As mentioned previously, the bulbospongiosus muscle is mostly resected, as when this is not done, some patients report inability to void when sexually aroused.

The cut edges of the urethra can be a source of ongoing bleeding after surgery, so thrombin-soaked gel foam is placed around the clitoris and urethral catheter before the bolster dressing is placed, with good effect against troublesome, ongoing bleeding.

### Labia majora fat

Some patients will have a large degree of adipose in the perineum that can be divided in the midline and then moved laterally to fill the labia majora, or it can be removed. If the amount of this fat is excessive, we tend to remove it, as there usually is plenty of left over adipose to make a plump, cushioned, and aesthetic labia majora.

### Clitoral sizing

The clitoris should be fashioned to be aesthetically equivalent to an adult cis female. In addition, the creation of a thorough and aesthetic labia major creates a clitoral hood that further recapitulates the ideal neovulva. The clitoris can initially be made small (i.e.: size of the tip of the 5th finger) but will often both retract and contract in size over time, possibly leaving a “too small” or even vanished clitoris. We prefer to make a slightly larger clitoris (i.e.: the size of the tip of the 4^th^ finger) to anticipate some shrinkage/contraction over time and to maximally preserve the erogenous nerves of the glans. Even though slightly larger at first, the neoclitoris ends up being thoroughly covered by the neoclitoral hood and seldom is protuberant. Rare cases of over protuberant clitoris can be revised in a small surgery after full healing has occurred (5).

### Vaginal Packing

An important step after completion of vaginoplasty is the placement of vaginal packing. We use Kerlix™ (11.4cm X 3.6m; Medtronic, formerly Covidien; Minneapolis, MN), as the thinner, standard vaginal packing used in other vaginal surgery is not bulky enough to fill the space. The Kerlix is thoroughly soaked in Clindamycin 1% foam and lubricating jelly. The packing is placed with Russian forceps to fill the cavity evenly, that is then sealed in place with the bolster dressing.

### Peritoneal vaginoplasty technique

For augmentation of the inverted penile flap canal, some centers have used the pelvic peritoneum to line the deep neovaginal cavity. This technique does add time, expense, and complexity, but can be advantageous for patients who have had puberty arrested and thus insufficient penile skin for the creation of a neovaginal cavity using the standard penile inversion technique (2). It also appears to be effective to salvage penile inversion vaginoplasty patients who have had marked shrinkage or obliteration of the vaginal cavity. Eventual epithelization of the neovagina is said to occur over some months (13).

This peritoneal vaginoplasty is similar to the penile inversion technique aside from the following differences:

1. The external genitalia are created as in the penile inversion vaginoplasty, including incising the future vaginal introitus, as described above.
2. The peritoneum is entered robotically. The pelvic peritoneum is incised, and a peritoneal flap pulled down to meet the vaginal introitus by upward, anterior, and posterior retraction, and sutured. Suture the caudal end of the peritoneal cavity.
3. Tack the anterior rectum to the peritoneum to prevent development of a rectocele.

### Visceral interposition vaginoplasty technique

In cases of congenital vaginal agenesis, revision of severe vaginal stenosis after previous inversion vaginoplasty, or grossly insufficient pe-nile length, sigmoid interposition or ileal vaginoplasty technique can be used, although we would generally favor a revision peritoneal vaginoplasty in these cases (14). Use of bowel might be advantageous for some revision cases because it provides a vascularized, tubular structure that provides its own mucous which can act as lubrication. It also may allow for easier inset into a previously operated field, as well as reducing the possibility of needing continuous dilations. Because of occasional patient complaint of foul-smelling mucous discharge after colon vaginoplasty, this technique is not often performed (2) and we avoid its use.

### Patient Satisfaction

Vaginoplasty has excellent outcomes among appropriately selected patients. Patient satisfaction can be maximized by extensive pre-operative counselling and setting realistic expectations for what the surgery can and cannot achieve. A pre-operative consultation first determines if the patient can undergo the surgery safely, and cope with the psychiatric/recovery/dilation demands of the surgery. Factors that can help determine if the patient is an appropriate candidate for surgery include personal and social stability, age of majority at the time of surgery, and a stable support system. Dissatisfaction, even to the point of surgical regret, appears to be increased by the presence of surgical complications (2). Therefore, it is crucial to address potential future self-limited and non-self-limited complications, and communicate effectively how those complications will be ameliorated should they occur (15). Requests for revision, usually to improve features of the cosmetic appearance can include reconstruction of the clitoral hood, formation of a more defined labia minora, or reduction of labia majora size/bulk (5).

### Post-operative care plans

We use postoperative clinical pathways (CPWs) to provide high quality care through aligning clinical practice with clinical best practices. CPWs seek to improve clinical outcomes while minimizing costs, and can improve overall healthcare costs, resource allocation, and length of stay (16). CPWs have been used in clinical practice internationally since the 1980s, and an estimated 80% of hospitals in the US had implemented CPW as of 2003 (16, 17). A 2012 Cochrane Review showed that CPW implementation consistently reduced in-hospital complications and improved documentation without increasing patient's length of stay or healthcare costs (16). In addition, a majority of studies reported that CPW's decreased in-hospital complications for surgical procedures (16).

We use a robust, standardized, 2-day inpatient CPW for all full vaginoplasties. In this way, order sets are standardized between patients, decreasing the potential for errors, and minimizing ad hoc orders placed for each individual. Nursing expectation for daily progress is also standardized. Patients who are not meeting expected milestones are identified early and receive extra diagnostic/therapeutic attention. Finally, our patient's understanding of the day-by-day hospital course is greatly improved. Patients are given individualized treatments, especially when encountering complications, but our CPW allows patient, physician, and nurses to be aligned as to each day's expectations and treatments. Because of the predictable safety profile provided by our post-operative CPW, most vaginoplasty patients are able to be safely discharged 2 days after the operation at our center.

Patients stop progesterone two weeks before surgery but are allowed up to 4mg/day of estrogen up until the day before surgery. Estrogen is held while in-patient, and restarted when the patient is ambulating reliably once home.

Patients with zero depth vaginoplasty are discharged home the day of surgery. Patients with a neovagina are admitted for an average of 48 hours after surgery, although some surgeons have good results with shorter hospital stays. They are placed on a standardized postoperative care pathway, with early nutrition and ambulation, ice off/on to the surgical area, and multimodal pain medication that includes muscle relaxers and NSAIDS, with use of narcotics only when needed. They are discharged home with vaginal packing and a urinary catheter in place and are to be seen in 5-7 days for packing/catheter removal. They stay on oral antibiotics until the packing is out.

At the time of packing removal, the patient is educated on how to dilate the neovagina and they begin a daily regimen of dilation. They are seen weekly for two more weeks and as needed after that.

## POST-OPERATIVE COMPLICATIONS

### Wound separation

**Figure 8:**
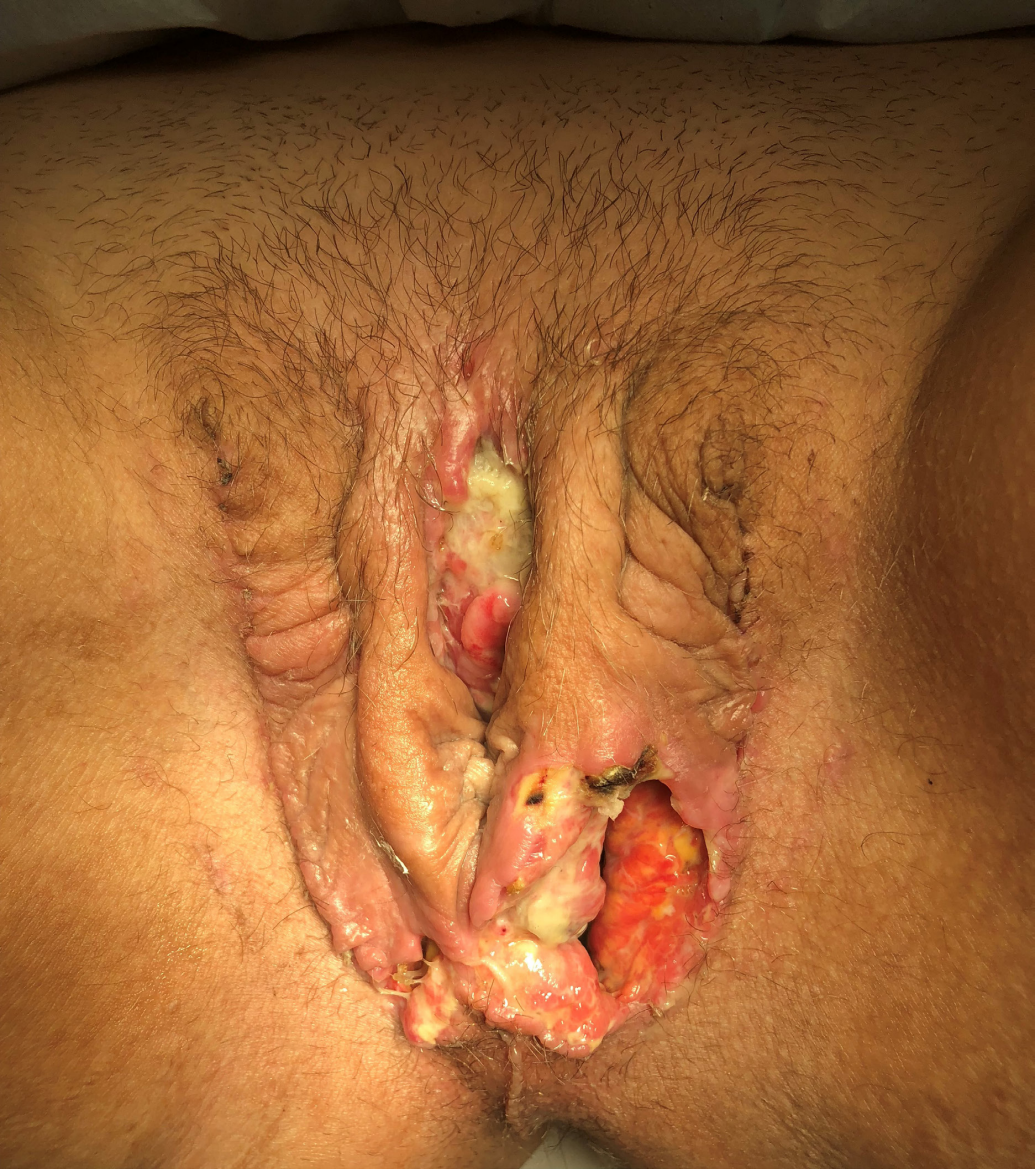
Moderate loss of the left inferior skin flap resulting in dehiscence of the suture line. This may be treated by wound debridement and irrigation and delayed primary closure. Note wound exudate over the clitoris is commonly present (as it is here), and usually resolves with time.

The most common postoperative complication is wound separation at the inferior introitus. Usually it is treated with dry dressings and expectant management. Minimal bedside debridement may be required, and rarely a thorough wound washout and delayed primary closure with nylon sutures may be needed (Figure-8).

### Vaginal stenosis

**Figure 9:**
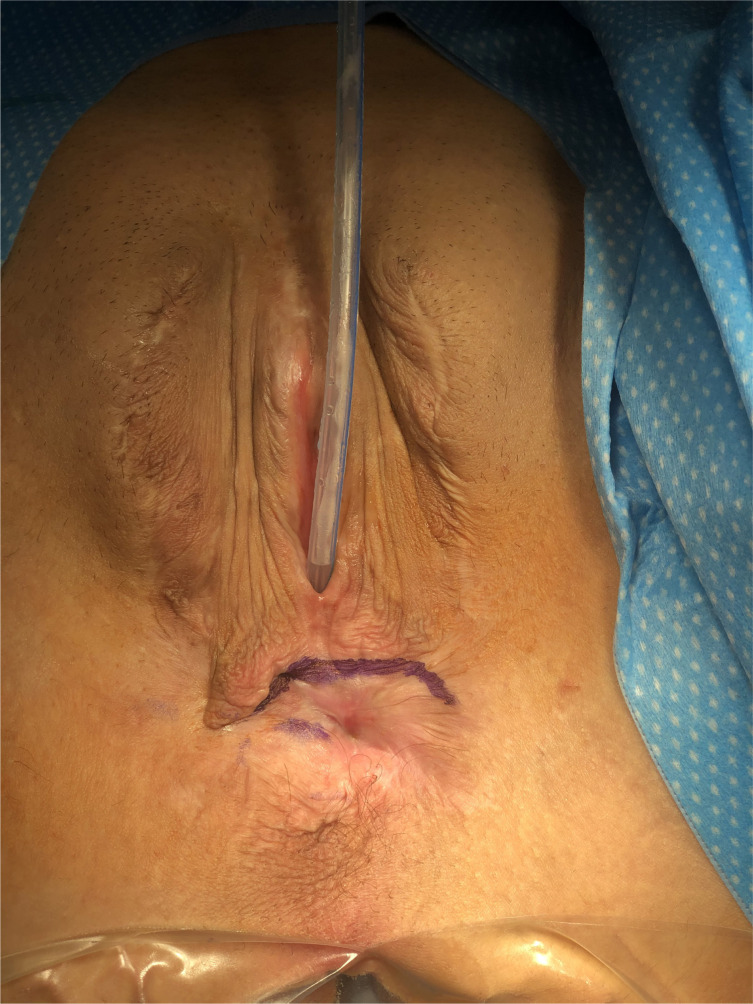
Total loss of the vaginal canal 10 months after vaginoplasty, in a patient with no previous signs of vaginal flap/graft loss. This patient has a notable history of healing other wounds very quickly and thoroughly and may represent a “phenotype” of patients with the ability to aggressively fill in surgical defects. Note the otherwise excellent appearance of the external genitalia.

The vaginal canal can become narrowed and stenotic, resulting in loss of depth and width (Figure-9). Vaginal stenosis is best managed by maintaining serial dilations, although an important minority of patients can still get vaginal stenosis despite diligent dilation. In case where only the introitus is narrowed, incising the “scar ring” and bringing in perineal skin using y-v plasty is often curative. In cases that need an involved revision vaginoplasty, skin grafts may be placed into incisions made into the neovagina. For more severe cases, revision peritoneal vaginoplasty or visceral interposition vaginoplasty may be required. In some patients, vaginal canal loss is due to inexorable healing of the vaginal cavity over time, despite all dilation efforts, which we hypothesize is more likely in certain patients with a history of exuberant scar formation, superfast healing, or other scarring-related disorders such as urethral stricture (5).

### Hematoma

**Figure 10:**
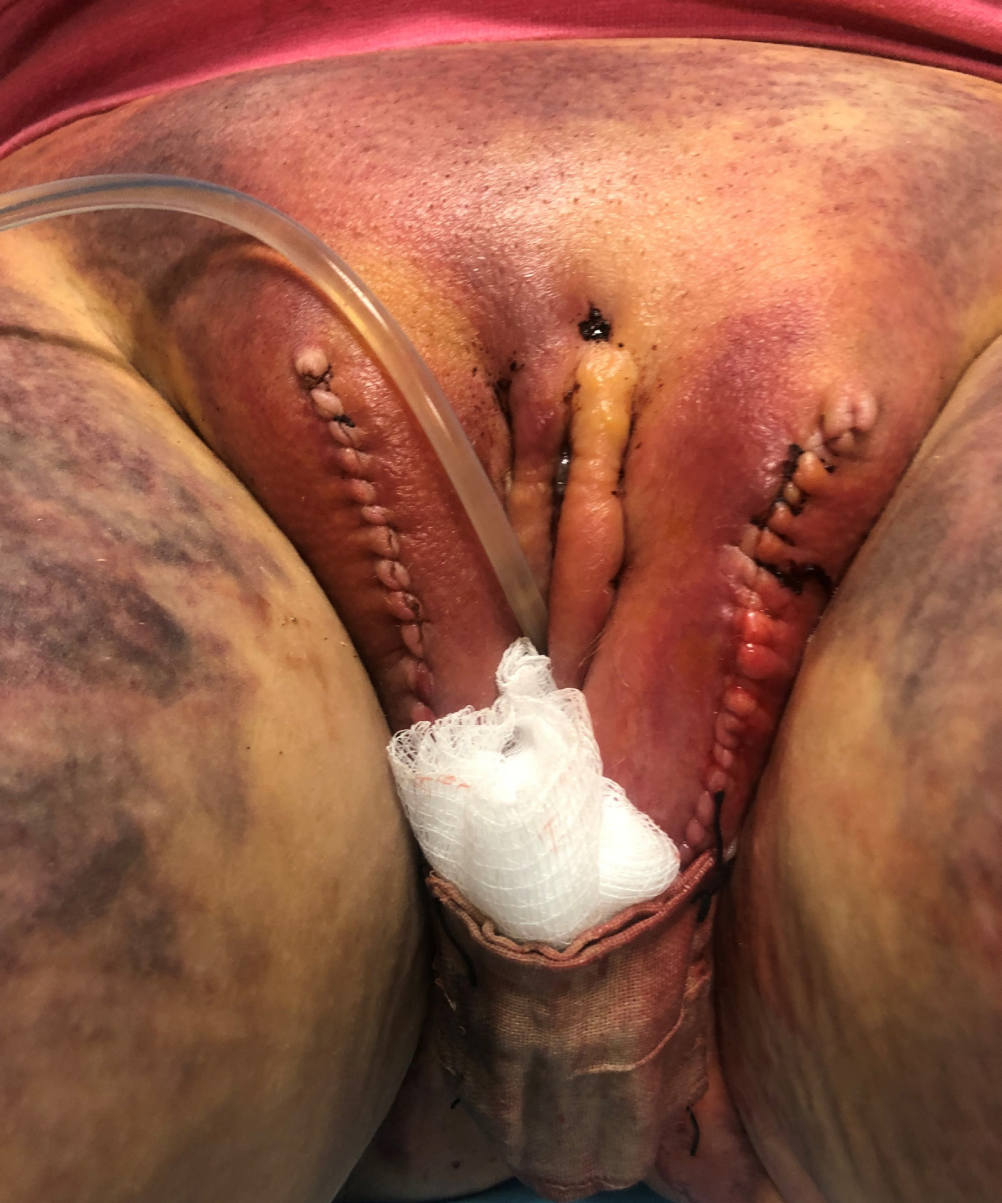
Severe post-operative swelling and likely hematoma. Note the distribution of bruising along the abdomen and thighs, conforming to the borders of Camper's/Scarpa's fascia.

Vaginoplasty surgery involves well-vascularized tissue and the cut edges of the corpora cavernosum, bulbar urethra, vaginal cavity and spermatic cord may bleed despite thorough surgical technique. Small postoperative hematomas are relatively common and may generally be observed. Large hematomas are uncommon and may require surgical exploration and drainage if they cause swelling that endangers the health of the flaps. (Figure-10).

### Rectovaginal fistula

Rectovaginal fistula is the most devastating complication for transfeminine genital surgery and can occur occasionally despite careful technique. Fistulas that develop in the absence of obvious rectal injury/repair may result from a occult vascular insult to the rectum (3). Other mechanisms include disordered healing of a known intraoperatively-repaired rectal injury or an undiagnosed rectal injury. Rectovaginal fistulas may occur early or late. Many fistulas are small and heal spontaneously, but fistula repairs may be necessary (5). Reparative surgery involves fecal diversion, resection of the fistula, and revision of the vaginoplasty (3).

## CONCLUSIONS

We and others endeavor to constantly evaluate and improve the steps of vaginoplasty surgery, and to investigate new concepts and materials in an ongoing effort to further improve outcomes. We have had good success with these techniques at our high-volume center, though more thorough investigation and quantification of patient outcomes is still needed. Ultimately, with continued innovation and sharing of improved surgical techniques, it may be possible to better standardize care and improve the aesthetic and functional outcome of this complex and increasingly common surgery.
